# Thrombin-Anti-Thrombin Levels and Patency of Arterio-Venous Fistula in Patients Undergoing Haemodialysis Compared to Healthy Volunteers: A Prospective Analysis

**DOI:** 10.1371/journal.pone.0067799

**Published:** 2013-07-02

**Authors:** James A. Milburn, Isobel Ford, Nicola J. Mutch, Nicholas Fluck, Julie Brittenden

**Affiliations:** 1 Department of Vascular Surgery, Aberdeen Royal Infirmary, Aberdeen, Scotland; 2 Division of Applied Medicine, University of Aberdeen, Aberdeen, Scotland; 3 Renal Medicine, Aberdeen Royal Infirmary, Aberdeen, Scotland; Institut National de la Santé et de la Recherche Médicale, France

## Abstract

**Background:**

Patients on haemodialysis (HD) are at an increased risk of sustaining thrombotic events especially to their vascular access which is essential for maintenance of HD.

**Objectives:**

To assess whether 1) markers of coagulation, fibrinolysis or endothelial activation are increased in patients on HD compared to controls and 2) if measurement of any of these factors could help to identify patients at increased risk of arteriovenous (AVF) access occlusion.

**Patients/Methods:**

Venous blood samples were taken from 70 patients immediately before a session of HD and from 78 resting healthy volunteers. Thrombin-antithrombin (TAT), D-dimer, von Willebrand factor (vWF), plasminogen activator inhibitor-1 antigen (PAI-1) and soluble p-selectin were measured by ELISA. C-reactive protein (hsCRP) was measured by an immunonephelometric kinetic assay. Determination of the patency of the AVF was based upon international standards and was prospectively followed up for a minimum of four years or until the AVF was non-functioning.

**Results:**

A total of 70 patients were studied with a median follow-up of 740 days (range 72-1788 days). TAT, D-dimer, vWF, p-selectin and hsCRP were elevated in patients on HD compared with controls. At one year follow-up, primary patency was 66% (46 patients). In multivariate analysis TAT was inversely associated with primary assisted patency (*r*= -0.250, p= 0.044) and secondary patency (*r* = -0.267, p= 0.031).

**Conclusions:**

The novel finding of this study is that in patients on haemodialysis, TAT levels were increased and inversely correlated with primary assisted patency and secondary patency. Further evaluation is required into the possible role of TAT as a biomarker of AVF occlusion.

## Introduction

Acute thrombosis of vascular access (VA) accounts for 20-25% of all hospitalisations of haemodialysis (HD) patients [1,2] which leads to considerable morbidity and cost to healthcare providers [3,4]. Coagulation activation has been implicated in the increased incidence of acute thrombotic events observed in patients on HD. Despite the use of heparin, or low molecular-weight heparins, a steady increase in thrombin generation has been shown to occur during HD [5–7]. In addition increased levels of fibrinogen and prothrombin fragment F1 + 2 (which reflects thrombin generation) have been found in patients on HD compared with controls [8,9].

Thrombin-antithrombin complex (TAT) is a sensitive marker of intravascular thrombin generation indicating activation of coagulation. Plasma levels in samples from both the VA and the systemic venous circulation are increased during dialysis and remain elevated up to 48 hours post-dialysis in patients [6,10]. In a small study TAT levels were found to be increased in patients on HD [11]. D-dimer is a degradation product formed when plasmin acts on cross-linked fibrin, and may reflect increased deposition of intra-vascular cross-linked fibrin, and/or up regulation of the fibrinolytic pathway. Increases in baseline D-dimer levels in patients on HD compared to healthy controls have been demonstrated and these levels have been shown to increase further immediately following dialysis [10,12]. Furthermore D-dimer levels are independently associated with the presence of ischaemic heart disease in HD patients [12,13].

While markers of the coagulation pathway have been shown to be increased both at the VA site and also in the systemic circulation [14] it is unclear whether there is an accompanying upregulation of the fibrinolytic pathway. Increased levels of tissue plasminogen activator (t-PA) have been demonstrated at the VA site, but not in the systemic circulation [15]. Erdem et al found no difference in t-PA levels between venous samples taken from healthy controls and HD patients [11]. Plasmin-antiplasmin complexes, and plasminogen activator inhibitor 1 (PAI-1) have been shown to be either increased or unchanged in patients on HD compared to controls [11,12,16–19]. Relatively few large studies have analysed both markers of the coagulation and fibrinolytic pathway, together in patients on HD compared to healthy controls and this area merits further investigation.

Endothelial dysfunction which has been shown to occur in patients on HD may also contribute to thrombotic events [20,21]. To date, there has been only been a limited number of prospective studies which have assessed the potential relationship between coagulation and/or endothelial biomarkers and VA patency outcomes. These have been limited by the inclusion of composite endpoints such as deep vein thrombosis and coronary events [22]. It is currently unknown if measuring cardiovascular biomarkers may help identify patients who are at increased risk of VA occlusion.

In this prospective study we aim to compare markers of coagulation, fibrinolysis and endothelial function in patients on HD compared to healthy controls and determine their relationship to arteriovenous-fistula (AVF) patency rates.

## Patients and Methods

Seventy consecutive patients on HD and 78 healthy volunteers were recruited into this observational comparative study over a 15 month period (Table 1). Patients were eligible for inclusion if they were established on HD and between 20 to 80 years of age. Patients were excluded if they had a history of haematological malignancy, a known bleeding diathesis or coagulopathy or concomitant warfarin therapy. Our definitions of patency are those recommended by the Society of Vascular Surgery and American Association for Vascular Surgery [23]. Primary patency is defined as uninterrupted patency without endovascular or surgical intervention being performed on the fistula. It is the interval from the time of access placement until any intervention designed to maintain or re-establish patency. Primary-assisted patency is defined as patency which is maintained by endovascular or surgical intervention. It is the interval from the time of access placement until measurement of patency. The secondary patency rate is defined as the time from formation of the fistula until the fistula is occluded or abandoned. Patients were followed up for a minimum of four years if their AVF remained patent or until the end of the secondary patency of the AVF.

**Table 1:** Baseline demographics.

	Controls (n=78)	HD-Patients (n=70)
Mean Age (Range)	48.9 (23-77)	59.0 (20-80)*
Sex Ratio (M:F)	28:50	46:24*
Reformed Or Current Smoker (%)	18 (23)	40 (57)*
Mean BMI kg/m^2^ (SD)	27.1 (4.78)	25.5^Ѱ^ (4.52)
	Diabetic (%)	12 (17)
	Coronary disease (%)	19 (27)
	Cerebrovascular (%)	4 (6)
	Peripheral Vascular (%)	3 (4)
	Any CV disease (%)	21 (30)
	Aspirin (%)	40 (57)
Clopidogrel (%)		5 (7)
Any Antiplatelet (%)		43 (61)
	Statin therapy (%)	35 (50)
	Erythropoietin therapy (%)	63 (90)
Mean Serum LDL-Cholesterol mmol/L (SD)	3.06 (0.84)	2.32* (0.76)

* P < 0.001, ^Ѱ^P< 0.05

CV − Cardiovascular disease, BMI − Body Mass Index, SD − Standard Deviation

Healthy control subjects had no history or clinical signs of renal, cardiac, cerebrovascular or vascular disease.

### Ethics Statement

Full ethical approval for the study was granted by the Grampian Research Ethics Committee. Each individual study participant gave full informed written consent prior to enrolment.

### Sample collection

It has previously been shown that there are differences in coagulation, fibrinolysis and platelet biomarkers between simultaneous samples in a single patient taken from the VA and peripheral veins [15,24]. In this case-control study similar sites of blood sampling were compared, namely the ante-cubital vein of fully rested patients in the HD and control group. A 21-gauge needle was used with a tourniquet applied to the upper arm. The cuff was removed once the first trickle of blood appeared in the first sample tube to prevent activation by stasis and the first 5 ml were discarded. Samples from the HD patients were taken immediately before the start of a single dialysis session and prior to administration of heparin. Unfractionated heparin was used as anticoagulant was used in all patients although they had not received any heparin for at least 48 hours. All patients were dialysed on synthetic Helixone™ membrane (Fresenius Medical Care, Notts, England) which is a polysulfone membrane. All membranes were high flux but differed in the surface area (m^2^) between FX 60 (1.4m^2^), FX80 (1.8m^2^) and FX100 (2.1m^2^). The samples were centrifuged at 2500 *xg* for 15 minutes, plasma was then transferred to a fresh tube and the centrifugation step repeated. The resulting platelet poor plasma was aliquoted and stored at below -70 ^o^C.

### Assays

**TAT** was measured using a commercially available ELISA (Enzygnost TAT micro, DADE Behring, Marburg GmbH, Marburg, Germany). The limits of detection were between 2 and 60 µg/l with an intra-assay co-efficient of variation (CV) of 5.7%.

**D-dimer** was measured using a VIDAS D-Dimer Exclusion automated quantitative test (bioMérieux, Lyon, France). The limits of detection were between 45 and 10,000 µg/l with an inter-assay CV of 3.5%.

**PAI-1 antigen** was measured using an in-house ELISA [25]. The assay has a limit of 30 ng/l and intra- and inter-assay CV of 7.2% and 5.5%.

**Plasmin-α_2_antiplasmin** was quantified using an in-house ELISA, as previously described [26].

**Von Willebrand factor (vWF)** antigen is a well recognised marker of endothelial dysfunction [26] and was measured by an in-house ELISA using polyclonal rabbit-antihuman vWF antibody and horse-radish peroxidise conjugated antibody (both Dako, Glostrup, Denmark) with an inter-assay CV of 7.8%.

**Soluble P-selectin** was performed using a commercially available ELISA kit (Human sP-selectin, BMS219/3CE, Bender Medsystems, Vienna, Austria). The limits of detection were between 2.19 and 140 µg/l with an inter-assay CV of 9.6%.

**High sensitivity (hs)-CRP** was measured using a commercially available immunonephelometric kinetic assay (BN ProSpec, Siemens, Tarrytown, USA) using Cardiophase hsCRP reagents. The limit of detection was 0.100 mg/l with an inter-assay CV of 3.1% and an intra assay CV between 0.5–2.1%.

### Statistical Analysis

Baseline demographics of HD patients and control subjects were compared using the Mann–Whitney U test or chi-squared test as appropriate. Where significant differences existed in the two groups (Controls to HD patients) multivariate linear regression analysis was used to adjust these comparisons for confounding factors which included age, gender, BMI, dyslipidemia and smoking history differences. Patency rates were calculated using life table analysis, log rank testing and Kaplan-Meier techniques. Correlation analysis was performed with Spearman’s rank coefficient and multivariate linear regression analysis. Data analysis was performed using SPSS version 20.0 (SPSS, Chicago, Illinois). A p < 0.05 was considered significant. Statistical advice for the study was provided by the Medical Statistics Unit, Department of Population Health, University of Aberdeen.

## Results

### Patient characteristics

Patient demographics are shown in Table 1. There was a higher proportion of males, increased age, increased incidence of dyslipidaemia, smoking and lower BMI in the HD patient group compared to controls. The cause of established chronic kidney disease was: chronic glomerulonephritis (n=20), diabetes (n=11), polycystic kidney disease (n=8), uncertain aetiology (n=8), obstructive nephropathy (n=7), renal artery stenosis/ hypertensive nephropathy (n=5), chronic pyelonephritis (n=4), congenital abnormalities (n = 3), Wegeners (n=2), bilateral nephrectomy (n=1), and renal tuberculosis (n = 1). All patients had been established on HD for a minimum of two weeks with a mean of 1013 days (Range 18–4370). The mean number of days the AVF had been patent for prior to sampling was 849 (Range 48–4488).

Haemostatic variables in HD versus controls

A linear multivariate analysis was performed adjusting for age, gender, dyslipidemia, BMI and smoking history.

### Coagulation

**Figure 1:**
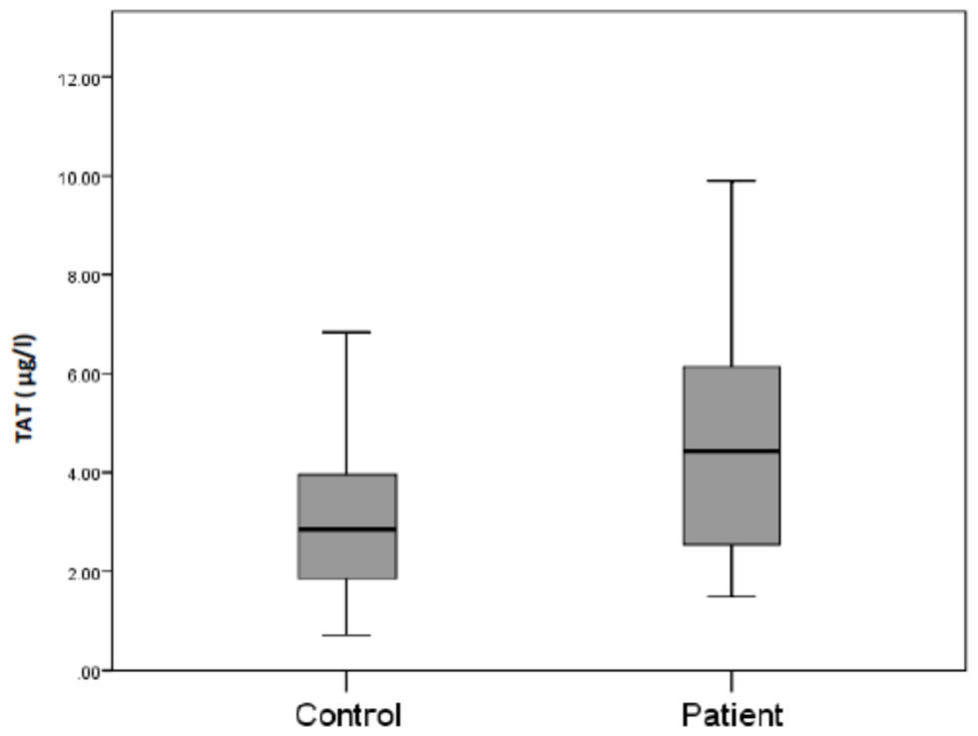
Plasma TAT concentrations in healthy controls and patients on HD. TAT was quantified by ELISA and the results expressed on a box plot showing the median (bars), minimum and maximum values (whiskers) and interquartile range (box).

TAT complex levels were statistically significantly higher in patients on HD compared to controls (median [interquartile range] 4.43 [2.53-6.17] versus 2.84 [1.81-3.82] µg/l p < 0.001) (Figure 1). TAT levels in patients on antiplatelet therapy were not statistically significantly different from TAT in patients not on antiplatelet therapy (median = 4.65 (IQR 2.45-6.31) versus median = 4.43 (IQR 3.56-5.33), p>0.05).

**Figure 2:**
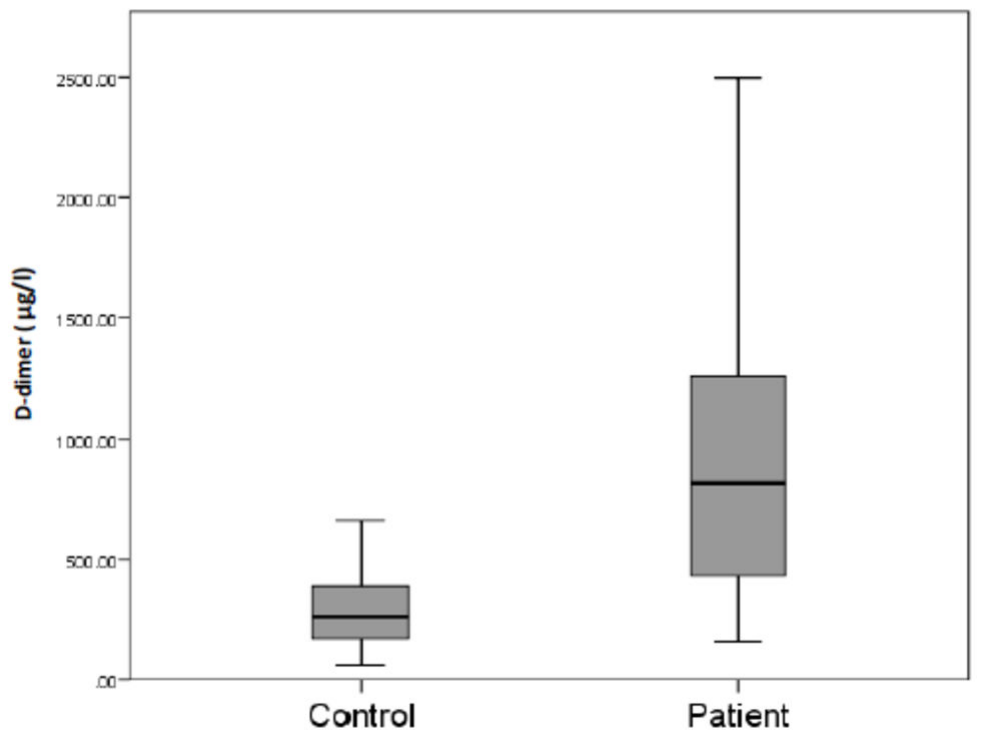
Plasma concentration of D-dimer in healthy controls and patients on HD. D-dimer was quantified by ELISA and the results expressed on a box plot showing the median (bars), minimum and maximum values (whiskers), and interquartile range (box).

Similarly, D-dimer levels were statistically significantly higher in patients on HD compared to controls (819.2 [428.6-1263.7] versus 265.5 [175.0-401.5] µg/l p < 0.001) (Figure 2). **Fibrinolysis**: There was no significant difference between plasma PAI-1 levels in patients on HD compared to controls (19.33 [12.23. -30.43] versus 23.22 [17.33-35.04] ng/l, p = 0.089). Plasmin-α_2_antiplasmin levels were also quantified in patients on HD and the control group, but in both cases were below the limit of detection (data not shown).

### Endothelial function

vWF antigen (P < 0.001), soluble P-selectin (p < 0.001) and hsCRP (p < 0.001) were statistically significantly elevated in the patients on HD compared to controls (Table 2).

**Table 2:** Markers of endothelial function in patients on HD and healthy controls.

Variable [Unit]	Controls (n=78) Median [IQR]	HD-patients (n=70) Median [IQR]
vWF [IU/ml]	0.878 [0.645-1.028]	1.50 [1.09-1.96]*
Hs-CRP [mg/L]	1.36 [0.67-2.69]	4.28 [1.59-8.99]*
Soluble P-selectin [µg/l]	24.7 [13.4-43.3]	40.4 [14.2-88.6]*

* P < 0.001 vs Controls vWF – Von Willebrand Factor, hs-CRP – high sensitivity C-Reactive Protein

### Patency Rates

#### Follow-up

**Figure 3:**
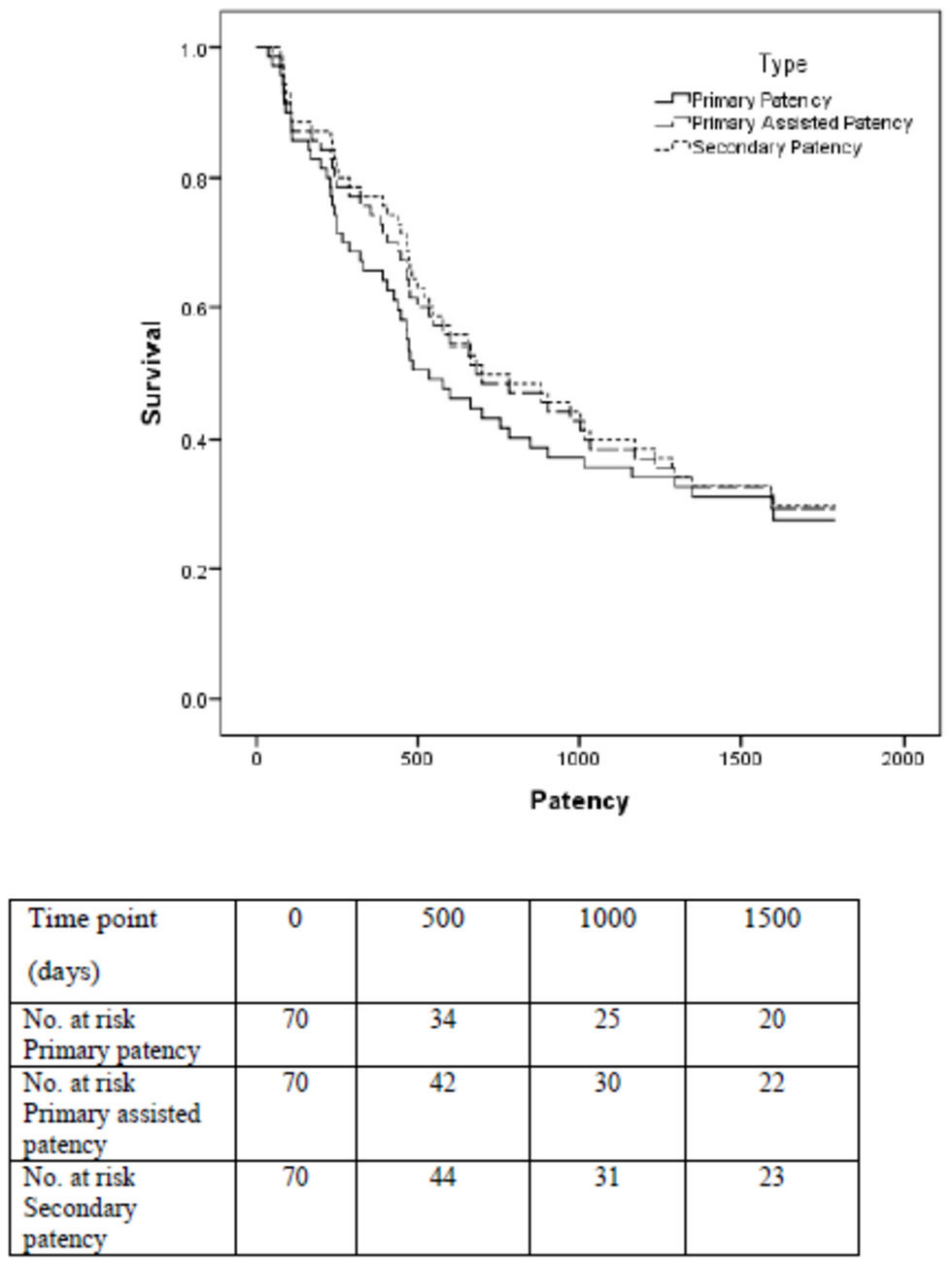
Cumulative survival analysis of patency for VA. The number at risk at each time point is detailed.

Median follow-up was 740 days (Range 72-1788). At 1 year follow-up, primary patency was 66% (46 patients), primary assisted patency 74% (52) and secondary patency 77% (54). Of these, 16 (21%) patients had interventions to maintain primary patency during follow-up. At a median follow-up of 740 days, 44% (31 patients) maintained primary patency, 49% (34) maintained primary assisted patency and 50% (35) maintained secondary patency. AVF survival is shown for primary, primary assisted and secondary patency (Figure 3).

#### Bivariate analysis

TAT showed an inverse correlation with primary patency (*r* = -0.341, p = 0.004), primary assisted patency (*r* = -0.296, p = 0.013) and secondary patency (*r* = -0.269, p = 0.024). hsCRP showed an inverse correlation with secondary patency (*r* = -0.242, p = 0.043). There were no other significant correlations with patency.

#### Multivariate analysis

The correlations between TAT and patency remained significant after adjustment for age, diabetes, smoking, history of cardiovascular disease, and number of days since AVF creation in a multivariate linear regression model. There was a trend toward an inverse relationship between TAT and primary patency (*r* = -0.238, p = 0.056). TAT was significantly inversely associated with primary assisted patency (*r* = -0.250, p = 0.044) and secondary patency (*r* = -0.267, p = 0.031). HsCRP no longer remained significantly associated with patency rates after adjustment.

## Discussion

This study has shown the existence of a procoagulant state in patients with established chronic kidney disease receiving HD, with increased levels of TAT and D-dimer compared to healthy controls. In contrast to previous studies we were unable to detect a significant difference in PAI-1 antigen levels in patients on HD compared to controls [17]. Previous studies have shown no differences in the levels of tissue plasminogen activator (t-PA) in patients on HD compared to controls [17]. In line with these findings we found negligible levels of plasmin-α2 antiplasmin complex in both the test and control group, indicating that plasmin generation was not stimulated in patients receiving HD. However, the significant increase in plasma D-dimer associated with patients on HD suggests ongoing activation of both the coagulation and fibrinolytic systems. A potential explanation for this finding may be that unlike D-dimer, plasmin-α2 antiplasmin complex is rapidly cleared from the systemic circulation and would be rapidly diluted by the high flow rate in the AVF. Therefore there may be a change in plasmin generation and plasmin-α2 antiplasmin complex at the fistula site which is reflected as a change in D-dimer in the systemic circulation.

D-dimer is excreted by the kidney and thus a rise in levels in patients with ESRD compared to healthy controls would be expected. Increased levels have also been shown to occur with advancing age, in patients with heart disease, infection, trauma, some cancers and in patients with a high rheumatoid factor [27]. While this suggests that a rise in D-dimer should be interpreted with caution, in this study the notable feature is that it occurred in association with a rise in the levels of TAT. We have confirmed the findings of the one previous small study which measured TAT in 26 HD patients and 22 healthy controls [11]. The novel finding of the present study is that TAT levels at baseline were inversely correlated with primary assisted patency and secondary patency. TAT is a sensitive marker of intravascular thrombin generation and to the best of our knowledge no previous study has looked at the relationship between TAT and VA patency rates. The utility of TAT measurement to aid the identification of patients at risk of acute cardiac events has been noted in studies involving patients with symptomatic cardiac disease including patients on HD [28,29]. This further suggests that the assessment of TAT may have a role in the identification of patients at risk of acute thrombotic events. The use of novel thrombin inhibitors, such as dabigatran, is increasing among patients with venous thromboembolism but also inpatients with atrial fibrillation and cardiovascular risk factors. In HD patients who are high risk patients for thrombotic events with raised TAT levels these drugs may be of potential value to prevent VA thrombosis and their role should be evaluated in future studies.

A previous study has assessed the relationship between D-dimer levels and patency rates and like our study showed no association [30]. However, Stolic et al, noted that plasma levels of D-dimer increased with time from fistula formation and at 1 year did show a similar level of correlation with patency rates. De Marchi et al prospectively studied 30 non-diabetic HD patients for 2 years and noted that PAI-1 and factor VII were independent predictors of fistula survival [31]. We found no increase in PAI-1 levels compared to healthy controls, although it should be noted that only 12 (17%) of patients in our study had diabetes.

The present study has suggested that alteration in the endothelial cell surface to a more pro-coagulant and/or inflammatory state occurs in patients on HD compared to controls as reflected by increased levels of vWF, P-selectin and hsCRP. Platelet adhesion to the vascular endothelium at high shear is mediated by multimeric adhesive vWF and plasma vWF is an established marker of endothelial activation. Increased plasma levels of vWF in patients on HD have been reported in previous studies [32]. Levels of vWF have also been shown to increase by 0.15 U/ml for every 10 years of age [33], but after applying this correction factor vWF was still significantly higher in the patients on HD compared to controls. However, we found no correlation between baseline vWF levels and patency rates. Sioulis et al, in a study of 54 HD patients, found that baseline vWF levels predicted an increased risk of a composite endpoint of thrombo-embolic events (including deep venous thrombosis, acute limb ischaemia and coronary events) at 15 months follow-up, but did not correlate it with access patency rates [22].

This is the largest study to assess venous samples for soluble P-selectin in HD patients and we have demonstrated elevated levels compared with controls. Soluble P-selectin predominantly reflects platelet activation, although it is also found in the Weibel-Palade bodies of endothelial cells from which it can be released upon activation. Thus, in the present study, the high level of soluble P-selectin in patients on HD may reflect both platelet and endothelial cell activation and could contribute to a prothrombotic state. It did not, however, show any relationship with patency rates.

HsCRP can lead to endothelial activation through induction of adhesion molecules, chemokines, neutrophil migration and microinflammation in the AVF [34–36]. CRP levels have also been shown to increase with age, but the differences observed between the groups in our study are larger than that which could be attributed to age alone [37]. We have shown that high levels of hsCRP are associated with a reduced VA patency rate, but this was not maintained on multivariate analysis. Chou et al, in a retrospective study showed a similar relationship [35]. We have previously shown hsCRP to be elevated in the VA of HD patients compared to simultaneous venous samples which may be a factor in local acceleration of VA thrombosis [24].

### Study Limitations

In order to enable a comparison with a control group it was necessary to study venous samples in both groups as the physiological conditions of a fistula cannot be reproduced in controls. The AVF samples as used in some studies of coagulation in HD patients are subject to turbulent blood flow and high shear stresses, which may lead to localised coagulation activation. We have previously shown that the levels of TAT, D-dimer, vWF, are similar in samples taken from vein and VA site, while hsCRP levels were increased in VA compared to venous samples [24]. The HD group and controls were not matched in terms of age, sex, BMI, lipid profile and smoking history and these were adjusted for in a multivariate linear regression analysis. Blood samples were not taken at the time of the patients’ fistula formation and the patients had been on HD for varying lengths of time, although the number of days since AVF creation was corrected for in the multivariate analysis. A further limitation was our inclusion of patients on antiplatelet agents which are known to affect platelet activation and thrombin generation. A Cochrane review supports the use of antiplatelet agents to prolong AVF survival [38]. We felt it would be unethical to limit the study to patients on HD not on antiplatelet therapy. We were, however, surprised that only 61% of our patients were actually receiving antiplatelet therapy. Importantly, we found no differences in the levels of TAT in patients who were or were not on antiplatelet therapy which is in keeping with the findings of previous studies [39,40].

## Conclusions

This study has confirmed the findings of one small previous study that TAT levels are increased in in the systemic circulation in patients with established chronic kidney disease receiving HD. We have shown an increase in fibrin deposition which may lead to a possible increased incidence of VA thrombosis. In particular, we have shown for the first time that levels of plasma TAT were inversely correlated with patency rates. Further large scale studies which measure TAT levels following the formation of a new fistula and serially thereafter are required to assess the role of TAT as a potential biomarker of vascular access occlusion. These studies would aim to establish an appropriate cut off value for TAT which would allow the identification of high risk patients such that appropriate targeted therapy and surveillance could be performed.
